# Preferences for Digital Smartphone Mental Health Apps Among Adolescents: Qualitative Interview Study

**DOI:** 10.2196/14004

**Published:** 2021-08-27

**Authors:** Robert Ribanszki, Jose Andres Saez Fonseca, Joseph Matthew Barnby, Kimberly Jano, Fatima Osmani, Soma Almasi, Elias Tsakanikos

**Affiliations:** 1 Thrive: Therapeutic Software London United Kingdom; 2 Division of Psychiatry Univeristy College London London United Kingdom; 3 Health Service and Population Research Department Institute of Psychiatry, Psychology and Neuroscience, Kings' College London, UK London United Kingdom; 4 Department of Psychology, University of Crete Crete Greece

**Keywords:** qualitative, adolescents, mental health, digital smartphone app, digital mental health, mobile phone

## Abstract

**Background:**

Mental health digital apps hold promise for providing scalable solutions to individual self-care, education, and illness prevention. However, a problem with these apps is that they lack engaging user interfaces and experiences and thus potentially result in high attrition. Although guidelines for new digital interventions for adults have begun to examine engagement, there is a paucity of evidence on how to best address digital interventions for adolescents. As adolescence is a period of transition, during which the onset of many potentially lifelong mental health conditions frequently occurs, understanding how best to engage this population is crucial.

**Objective:**

The study aims to detect potential barriers to engagement and to gather feedback on the current elements of app design regarding user experience, user interface, and content.

**Methods:**

This study used a qualitative design. A sample of 14 adolescents was asked to use the app for 1 week and was interviewed using a semistructured interview schedule. The interviews were transcribed and analyzed using thematic analysis.

**Results:**

Overall, 13 participants completed the interviews. The authors developed 6 main themes and 20 subthemes based on the data that influenced engagement with and the perceived usefulness of the app. Our main themes were *timing*, *stigma*, *perception*, *congruity*, *usefulness*, and *user experience*.

**Conclusions:**

In line with previous research, we suggest how these aspects of app development should be considered for future apps that aim to prevent and manage mental health conditions.

## Introduction

### Background

The rise in common mental health issues among adolescents is a distressing trend. The World Health Organization has estimated that 20% of adolescents experience mental health conditions, and most of them do not receive or seek appropriate diagnosis and care [1]. Addressing this concern is an essential component of the current global mental health agenda [2]. Innovative solutions delivered by mental health apps (MHapps) could represent a feasible solution to tackle this issue.

There is already a plethora of mental health mobile apps available to adolescents, and the increasing use of smartphones in this group might make these apps more acceptable and accessible [3]. Studies have also suggested that complex app-based mental health interventions for adolescents are feasible. For instance, in one study, cognitive behavioral therapy in the form of SMS text messages was considered useful by 75% of the participants [4]. Moreover, significant engagement with MHapps has been shown in the past, with nearly three-quarters of adolescents completing more than 80% diary entries over the 1-week intervention period [5]. MHapps also have the potential to reduce barriers to face-to-face help seeking, including stigma and distress about discussing one’s own mental health [6]. This aspect of MHapps may be appealing to young people, given that most adolescents would not seek or pursue help with respect to mental health through traditional routes [7].

Adolescents’ familiarity with mobile devices suggests that technology-based approaches would benefit them [8]; however, it is crucial to understand how best to tailor digital interventions to make them the most appealing. Tucker and Goodings [9] identified three themes that characterize most current MHapps, as follows: stress-inducing or stress-reducing apps, apps for configuring the body in space, and digital self-care apps. However, apps targeting adolescents likely need to expand into other areas such as positive focus, customizable features, human-human interaction, and easy access [3]. In terms of help-seeking preferences, adolescents have also expressed a desire for web-based, accessible information and health interventions, which are all technology-based needs rather than needs that can be met via in-person, telephone-based, or paper-based services [10]. When considering communication with providers, adolescents preferred email or text over video communication [11].

Despite their familiarity with digital technologies, engagement is generally low and evidence on the usefulness of concrete features is still scarce [12,13]. In addition, engagement with MHapps seems to vary independently from the presence of evidence-based features [14]. This raises questions about the clinical effectiveness and safety that undermine trust in both users and providers [15]. Furthermore, high dropout rates are generally associated with poor user experience (UX), whereas the specific components of engaging MHapp design are yet to be determined [16,17].

### Objective

In this study, we seek to identify the key preferences and attitudes of adolescents that future digital mental health interventions may need to take into account to successfully reach this population. For this purpose, we used the *Thrive* mental health app, which has an established evidence base [18,19], to explore adolescents’ perceptions of the potential usability of such a tool in their everyday life. Using a qualitative design, we gathered feedback from a sample of adolescents through face-to-face interviews. Specifically, our goal was to detect potential barriers to engagement and to gather feedback on the current elements of app design regarding UX, user interface (UI), and content. The long-term aim of this exploratory study is to provide the foundations for creating digital interventions for adolescents that are equally driven by clinical rigor and UX to help retain engagement.

## Methods

### Ethics and Preregistration

This research study was approved by the Roehampton University ethics board (reference number: PSYC 18/306) and preregistered as a qualitative protocol (reference number: TCYP171110). All methodologies adhered to the protocol unless stated otherwise.

### Participants and Procedure

We recruited a total of 8 male and 6 female participants (N=14). Overall, 11 participants were recruited from a local secondary school attended by approximately 1300 students at the time of the study. Students were made aware of the study through combined advertisements in the school. For interested students, the researchers gave a talk that outlined the overall purpose of the app and explained that the study was trying to understand what features of digital MHapps were most useful to keep students engaged. Furthermore, 3 participants were recruited through their parents who were also users of the app and knew about the study. These 3 participants were enrolled after an introductory conversation with the researchers, explaining the details and the purpose of the study.

Participants in the school were asked to get in touch with the lead teacher to express their interest in participating in the study. The teachers assessed whether the students adhered to the inclusion criteria to be a part of the study. Researchers then got in touch with the lead teacher to schedule a meeting in person on school grounds to interview the participants. Participants who were recruited via end users were interviewed over the phone. Consent forms for participants, parents, and teachers were sent out and returned via email. The information sheets were sent via email. The participants were interviewed as soon as possible after they had given their consent forms and had used the app for at least 1 week.

**Figure 1:**
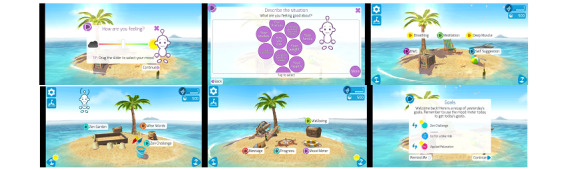
Screenshots of the Thrive: Feel Stress Free app. From top left to bottom right, this figure shows the mood meter, mood bubbles, first menu, second menu, third menu (all menus are accessible from the main screen and have an island theme), and goal menu.

All participants were asked to use the *Thrive: Feel Stress Free* app (Figure 1) for 1 week as much as they liked. Participants were asked to turn on their notifications in the app; however, this was not enforced as a strict inclusion criterion.

### Sample Size and Theme Saturation

Although our target sample size was 30, recruitment was stopped after 13 participants had been interviewed. The reason for this decision was twofold:

1. Owing to unforeseen barriers (school examinations, holidays, and teacher availability), our initial recruitment strategy resulted in 13 interviews, and another round of recruitment would have been needed to reach our intended sample size.
2. At this stage, we decided to review our sample size estimate by assessing theme saturation in our data.

We defined saturation as “the point during data analysis at which incoming data points (interviews) produce little or no new useful information relative to the study objectives” [20]. Using an approach recently refined by Guest et al [20], we set out to estimate how many additional interviews might be needed to reach theme saturation in our data. As most novel information is seen early in the coding process and follows an asymptotic curve in qualitative data sets [21], it is possible to use the occurrence of novel codes in each subsequent interview to estimate the slope of this curve and make an informed decision about a new recruitment target by systematically coding available interviews. However, after the coding of interview 9, it became apparent that saturation had already occurred and no further recruitment was necessary. To assess the saturation, interviews were coded by 2 of the researchers—JASF and JK—none of whom took part in interviewing participants or transcribing interviews.

### The App

We chose the Thrive app for this study because we considered its design and UX elements as good examples of the state-of-the-art development principles in the digital mental health field. Moreover, one of the authors (JASF) was involved in the development of the Thrive app, which allowed us easy access to the app for the purpose of this study.

After opening the app, participants were guided through a short mood assessment and thought training exercise, which allowed the app to create a customized cognitive training plan for the user. The recommended exercise modules (and others) could be accessed after the assessment phase was complete. The included modules were a combination of guided relaxation techniques and guides that provided further background and recommendations related to the given feature, such as meditation, breathing, or self-suggestions. Exercises were explained in detail, and the users were guided through the entire process when practicing each module. Next to the cognitive training features, participants were able to play games, such as Zen Garden or word puzzle, aimed to provide a more engaging UX. When finished with the modules, the app also provided participants with an overview of their progress where they could track their mood, practice, and goals. Examples of these steps are presented in Figure 1.

### Data Collection

Data were collected between January and July 2018. Owing to unforeseen delays, such as school examinations, holidays, and teacher availability, this was substantially longer than reported in the protocol. Eligible participants were between the ages of 11 and 18 years, owned a smartphone, used it frequently (more than 1 hour a day), and did not have an existing diagnosis of a mental health condition.

During the interview, no one else was present aside from the participant and the researcher, either in person or over the phone. We did not interview participants as a focus group because of the difficulty in organizing this at a convenient time.

The sessions were guided by a semistructured schedule (Multimedia Appendix 1). The discussion began with participants’ overall impressions of the app. For example, “what bits of it [the app] were useful if any” or “which bits of it [the app] did you dislike?” The schedule then proceeded to more specific questions if not covered in the overall broad questions. For example, “what did you think of our avatar” or “what did you think of the journal?” This was not pilot-tested before commencing the interviews but was constructed by consensus between the authors.

Participants were asked when they would engage with the app during the day, if at all, and at which location this took place. We asked which barriers and facilitators led them to use the app less or more frequently. In addition, we asked participants to list what they would change about the app to make it more interesting. The participants led the conversation though the interviewers ensured that the participants were prompted to specific topics if previously missed. Each interview lasted approximately 20-40 minutes. No repeat interviews were conducted. The interviews were audio-recorded and transcribed verbatim by SA, FO, RR, and JMB.

### Analysis

The interview extracts were analyzed using inductive thematic analysis [22]. First, interview transcripts were read carefully by 3 researchers (RR, JASF, and KJ) to identify meaningful ideas relevant to the research topic. On the basis of this, an initial list of relevant concepts was generated. Second, short segments of the data, dealing with similar issues or concepts, were identified and grouped together using provisional codes. At this stage, researchers coded each transcript on their own and could use different codes for any single data segment. Third, codes were discussed and cross-referenced between the researchers and collated into a common framework that allowed for candidate themes to emerge. Finally, upon review, candidate themes were grouped into main (or meta) themes that formed coherent meaningful concepts across the texts. The main themes and subthemes were then reviewed and refined using the original transcripts and linked to participant quotes to ensure that the final themes indeed formed a coherent pattern across the whole data set. Our analysis resulted in 6 main themes and 20 subthemes (Textbox 1).

**Main themes and subthemes developed by the authors based on the data on the experience of using a digital mental health app.**

- **Timing:** Daily schedule, exercise length
- **Stigma:** Family and friends, visibility, independence, framing, avoidance
- **Perception:** Trustworthiness, seriousness, skill versus quick fix
- **Congruity:** Design versus content, complexity, user journey
- **Usefulness:** Control, labelling, prompts, past experiences, valued features
- **User Experience:** Gamification, personalization

## Results

### Overview

One participant was not available for the interview, so our results included 13 interviews in total. Most participants were White and British (12/13, 92%), and 1 participant self-identified as being on Asian descent.

We labeled our main themes as follows: (1) timing, (2) stigma, (3) perception, (4) congruity, (5) usefulness, and (6) UX. Although our aim was to treat these categories as separate, they are nevertheless closely intertwined concepts with inevitable overlaps. The subthemes are referenced by their numbers in parentheses in Textbox 1.

### Timing

All participants emphasized the importance of time constraints when engaging with the app. Participants often described their daily schedule as *busy* and *stressful* where they have to *get on with their work*. Under these circumstances, using the app often felt like an extra task where “you do have to make a conscious effort to go in” (Participant 5)*.* This routine usually limited participants to only engage with the app at home, particularly before bed when there is nothing else left to do; however, this also led to its own set of issues. It was frequently mentioned that the app was *not being in sync* with this kind of schedule:

Generally, I was doing it more in the evenings and by then I couldn’t, it was giving me like tasks to go out and go for a walk and things and I couldn’t because it was dark outside.

Participant 4

The pressure of having a busy schedule also made exercise length an important question. In general, shorter and easily accessible exercises were more appealing, where users could simply log in and *check on themselves* by noting down their mood or completing a quick task. In this context, routine mood screening measures before accessing any particular exercise and longer tasks were usually seen as barriers to engagement. Participants were already conscious of these time restraints even before deciding to log in and knowing that they had enough time to complete a task was seen as an important determinant of engagement:

I didn’t want to go on the app for a bit because I just thought ‘this is going to take ages now, as if I knew I could do one that literally lasts like 4/5 minutes then I’d just click on that.

Participant 6

### Stigma

Mental health–related stigma was one of the most frequent themes in our data, and it is likely to have a significant impact on engagement. Stigma primarily emerged through labels such as *weird, uncomfortable, private matter,* and *ashamed*, which referred to feelings of embarrassment and vulnerability associated with using a mental health app. Even though these issues were common to all, only 1 participant mentioned encountering any negative remark:

There was one goal to go out for a walk and going out randomly for a walk is a bit weird so you know I was explaining it to them and they found it a bit silly at first but then as I was going through it with them and explaining how it worked they found it more interesting...I mean there was one member of my family that still thought of it as still a bit gimmicky.

Participant 4

As this negative comment was unique in our data, we saw stigma as already internalized, which was brought to the surface by certain situations when using the app.

Most frequently, users were cautious about openly using and/or talking about the app among their family and friends and in public, such as on public transport where the *risk* of being seen and labeled was highest. The presence of other people was also a barrier to performing various exercises, such as deep breathing or closing eyes, as these activities were seen as uncomfortable in public. In general, mental health was thought of as a *private matter* that belonged *behind a closed door.*

Thinking of mental health as a private issue also made the app more appealing in other ways. Participants perceived that it gave them more control over their issues without having to rely on other people:

I know a lot of teenagers who maybe wouldn’t want to go to a counsellor or would be ashamed of going to a counsellor and in this way you’re kind of helping yourself in your own way.

Participant 12

Participants also wanted to avoid being seen as a *downer* who complains about being stressed and preferred relying on an app rather than risking social rejection.

Participants were also concerned about their self-perception when using the app. Many of them took issue with the term *mental health app* and suggested other labels with less *loaded* connotations. Good examples of such alternative terms were stress reduction or well-being (app):

I wouldn’t say I have something as like a mental health issue. I think it is more just being stressed, I think people wouldn’t look at it the same way.

Participant 13

Some participants felt that having any issues is in itself problematic and question the utility of engaging with them:

I thought, like, sometimes stuff you didn’t really realise you’d thought about was there and it’s a bit depressing to think about that.

Participant 6

### Perception

Engagement was also closely tied to the way participants perceived the app, most frequently, to whether it was perceived as trustworthy. However, it was not always clear what trustworthiness meant. Some participants defined it as *professional*:

It definitely seemed quite slick and professional rather than a new app. It seemed like it had a backing to it.

Participant 6

Although others described it in contrast to other apps on the market:

It didn’t say from the very start that this is some meditation type hippie app...because you know there’s other apps out there that come across from the very start as that kind of thing. And you just think ‘they’re not going to work.’

Participant 7

Participants also highlighted a fine middle ground between being too serious on one hand and childish on the other. Most of them picked up on *playful* design elements, which were generally considered childish, even though the app was designed with adults in mind. One participant even reflected on this sensitivity to being patronized:

Teenagers always get funny about things being childish. Especially like slightly younger 14-year olds. They wouldn’t want to feel like it was for children at all.

Participant 9

Conversely, participants also did not like the *medical* label attached to the app, which may be perceived as too serious:

I think for lots of people who aren’t necessarily that comfortable talking about mental health and all that would feel almost slightly uncomfortable by...when they are gonna download it and says it is medical. You don’t like feeling like that; like they have got a problem.

Participant 8

Following design, a common element was the way participants viewed the role that an app like this should play in their daily life. When asked about how they would describe it to a friend, a “use it only when you feel stressed” approach was overwhelmingly popular:

I described it as like a self-help, meditation app that you could use in stressful situations.

Participant 1

Although the app itself was created with structured skill building in mind, the default perception was that it should be used as a quick check-in tool or quick intervention when one is anxious or needs immediate support.

### Congruity

Congruity describes some of the key areas where the design of the app (both UX and UI) proved to be confusing or working against its intended purpose. A common experience that seemed off-putting to users was when the design of various features was not in line with its actual content. The most frequently mentioned examples were the relaxation exercises; a participant illustrated this as follows:

In comparison to the really slow breathing and stuff, a fast-moving thing (background) just seemed really big at the time.

Participant 6

Along the same lines, participants also found looking at a screen problematic right before going to sleep—one of the most popular times to use the app—as its negative effects may do more harm than good and missed the option of a voice-only session for this situation.

In addition, on the UX side, participants preferred more guidance from the app, as without it—or without preexisting knowledge of mental health and therapeutic techniques—some sections of the app proved too complex:

I found the meditation section kind of overwhelming at first. Because there are so many options. And I found I didn’t know quite which one to start with and then carry on with.

Participant 12

These types of problems can be overcome by clear signposting and well-designed user journeys; however, for some participants, this aspect has proved confusing as well:

...when you log in it’s not as straightforward to follow the instructions or like when you first log into it.

Participant 1

### Usefulness

Going beyond content and design, the usefulness theme refers to common patterns in participants’ subjective experiences that either contributed or hindered effective engagement with the app. Many pointed to the positive effects of having a sense of control when it comes to mental health. Therefore, the fact that they were able to *do something* about their problems was in itself beneficial:

So, it felt like I was like actively going out and helping myself...rather than me just thinking “oh my goodness I’m just swamped,” I’m actually making an effort to climb out of this mess.

Participant 4

Along the same lines, exercises were also most beneficial when users clearly understood their purpose and saw their progress:

Because you can kind of start to see if anything is like changing. If you know what it’s trying to achieve and how it’s trying to achieve that, then you can kind of measure how it’s working. So, yeah I think it was useful to know what it’s doing and what it’s trying to help you think about.

Participant 2

Although having control over a problem was generally seen as a good thing, predefined situation labels were generally seen as a step too far. Common complaints were that these labels were often simply *wrong*, too *restrictive,* or *not accurate enough,* and although predefined labels were also useful for many, the participants suggested adding the option of having their own labels:

I did like the idea, but I felt if we were allowed to write our own responses instead of choosing one it would be more powerful.

Participant 9

Having the choice of selecting from predefined automatic thought labels also proved problematic for some users, especially if they were already in a negative mood:

...when I was in a bad mood it would ask me what I was thinking before I hit that bad mood and some of them were quite extreme. So, I latched onto the quite extreme version of what I was thinking.

Participant 4

Others also described similar experiences related to mood questionnaires, pointing to a situation where the app might even cause extra distress, by highlighting existing problems:

I might not even realise I’d been thinking about, like worrying about doing this and that. Not really realising, more subconscious. And then I click on it and I realise I actually have been doing that, and it makes it worse as it explicitly says it.

Participant 6

Some users also developed an association between their low mood and engaging with the app, which over time even amplified certain negative states:

...when you’re in a bad mood and you just kinda don’t say explicitly you don’t necessarily stay in that mood, but when you click on the app, like a dark cloud or whatever it is, I then just feel like down.

Participant 5

Users’ past experiences with certain exercises, such as meditation, proved to be a strong contributor to the kind of features they liked and visited frequently. This also underlines the appeal of activities where users know what and why they are doing. Beyond meditation exercises, the most valued features were reminders, relaxation or sleep exercises, the mood tracker, and the peer support or communication function.

### UX Theme

This final theme highlights some of the emergent contradictions and alignments between current trends in app design and participants’ subjective experiences in 2 key areas: gamification and personalization.

With regard to gamification, users saw games in this context as either neutral or counterproductive, although there was one participant who suggested that games should be more competitive rather than *calming*. Outside of the concrete games, users also did not make much use of other *soft* gamification features, such as the point system, whereby users were able to unlock new achievements and earn credits. This progression system, without real tangible rewards, did not make sense to the participants. Conversely, specific features of progression systems, with which participants were able to unlock new content based on their achievements, were generally seen as useful in creating a sense of progress and contributing to engagement:

The games in the app are definitely more fun and enjoyable because I can feel better benefit from them whereas with other puzzle games I just get frustrated if I can’t do it. Whereas with on the app, I think, if I can’t do it, I just keep going. There is no frustration in it.

Participant 2

Participants valued the ability to personalize their own experiences by setting their own background to creating their own character. They also wanted the opportunity to send personal messages to one another and did not see much value in sending or receiving prepopulated messages:

So it would be quite nice to have that feature where you could talk to people. Sort of like a news column, where you could put your queries or ask the app and you could put in your questions and they answer back and you can do it vice versa.

Participant 10

## Discussion

### Principal Findings

We aimed to understand adolescents’ preferences when engaging with digital mental health interventions using semistructured interviews. Using these data, we developed 6 main themes and 20 subthemes that captured distinct aspects of adolescents’ experiences with the Thrive app. Overall, most themes corresponded well with previous research in the field [7]; however, we also gained new insights for further exploration.

Adolescents saw the app as helpful. Many expressed that simply having access to an MHapp was reassuring in itself and proved beneficial in increasing their sense of self-reliance and containing negative moods. Time was a major factor in terms of engagement. Most participants reported having a busy schedule and preferred using the app in the evenings before bed or just for quick *check-ins* during the day. Preferred features also corresponded with evening use and underscored results from previous studies highlighting the need for brief and easy-to-access features [23].

In terms of specific features and design, participants highlighted the importance of clarity in both their user journey and available information, which was also emphasized in previous studies [7,23]. Features seemed the most popular and engaging when either users already had experience with similar exercises, such as meditation, or when the purpose and goals of a given feature were clearly defined. We believe these preferences suggest that clarity of information likely has a direct impact on effectiveness by providing users with a clear mental map through which they can progress.

In contrast to previous studies that suggest reward and progression systems as a way of facilitating engagement [23,24], we found a clear distinction between helpful and unhelpful progression systems. In our sample, simple leveling or point systems were not meaningful to participants if they were not tied to tangible rewards. Similarly, games did not facilitate engagement as users deemed them irrelevant in this context. This was unexpected given the age of the population and previous research indicating good acceptability of games in this context [25]. Most participants endorsed the ability to unlock new features and levels that provided them with access to new content and exercises.

Stigma emerged as a hidden but important barrier to engagement. Participants often expressed embarrassment and feelings of weakness related to mental health. This concern, although not surprising given the associated stigma in the field [26], led many participants to use the app only in private and question how the app was branded and framed. Participants’ reluctance to use the app when traveling is especially problematic when we consider that this could be the most obvious opportunity to find the time to engage with a mental health app. As a solution, less conspicuous designs were suggested.

Other barriers mentioned by participants also converge with those of previous research pointing out the stress-inducing potential of these apps [9]. Specifically, some participants saw prepopulated questions about their feelings as stress inducing. Others also felt that engaging with negative thoughts, rather than ignoring them or simply letting them pass, was not desirable. However, this seems to contradict previous findings that praise apps for their ability to increase emotional awareness [27,28]. This avoidance may hint at a difficulty in enduring or accepting any amount of distress. This may stem from an assumption that one should not have to encounter anything that may be distressing or difficult in life. We believe that this potential unwanted effect of MHapps deserves further investigation, as bringing negative thoughts into awareness is a fundamental aspect of cognitive behavioral therapy and accepting this initial *dip* in mood is a prerequisite for effective engagement [29].

Along the same lines, the majority of participants used the app only for checkups and as a quick stress reduction tool in acutely stressful situations. This pattern of engagement is in stark contrast to the skill-based approach to improving mental health where users engage with MHapps to acquire the skill of managing their distress on their own and self-soothe. Although acute stress reduction can indeed be beneficial in certain situations, excessive reliance on this may increase dependence on an external source of soothing rather than reducing it. We see this mismatch in user attitudes and intended use as one of the key points to address in the future if MHapps are indeed to become scalable additions to therapy.

Finally, although previous studies often emphasized the importance of customizability [3] and this also emerged as a desirable feature in our sample, given that time seems to be one of the most important factors influencing engagement, this finding should be approached with caution. Participants may express their desire for customizability in an interview situation, but in reality, they may respond better to clearer design, short interactions with the app, and easy access.

### Limitations

Participants were only provided with access to the app for 1 week before the interview, which might have influenced the depth and detail of their experience and limited our conclusions. However, given that our main goal was to detect immediate, noticeable features that were liked or disliked, and that we reached theme saturation in our analysis, we are confident that this time frame was sufficient to address our question.

Another potential source of bias is the small sample size and the convenience sample. This may mean that our participants may be overly similar in certain ways, having similar backgrounds and preferences, which could distort our findings. Collecting background information from participants would have enabled us to reflect on this aspect in more detail. Moreover, this information would have also helped us to adequately contextualize our results. Although our focus of this qualitative paper was to get a *sense* of how adolescents may view a mental health app, more background on demographics and other participant characteristics would have strengthened the interpretation of our findings.

Finally, 3 participants were known to the experimenters. This may have caused some bias in the study as participants may have been interested in the app from the start and had an incentive to speak favorably. Although it was made clear that they would be anonymous and their responses recorded by a member of the research team not known to them, it is still reasonable to assume that there may be some bias regarding their experience of the app. However, we did not observe any differences between the responses of the participants along these lines.

### Conclusions

We identified 6 main themes and 20 subthemes that captured distinct aspects of adolescents’ experiences with the Thrive app. Overall, participants preferred convenient, clear, and easy-to-access features that they could use on an ad hoc basis. They saw the app as a potential way of calming themselves when needed rather than as a tool to learn to manage their mental health. Contrary to our initial assumptions, some specific design elements, such as the outlook or games, were seen as childish or useless. Furthermore, stigma emerged as a major barrier when engaging with the app. This again points to a mismatch between the intended purpose of the app and the way it is perceived by its potential users. Future studies with larger sample sizes are needed to dissect the specific preferences of specific user groups and to outline concrete ways to address these barriers in the digital mental health field.

## Appendix

Multimedia Appendix 1 Semistructured interview schedule.

## Abbreviations

MHapps: mental health apps
UI: user interface
UX: user experience
