# Supplementary material for: Preferences for Digital Smartphone Mental Health Apps Among Adolescents: Qualitative Interview Study
Source: JMIR Form Res. 2021 Aug 27;5(8):e14004. doi: 10.2196/14004 (PMC8433947; doi:10.2196/14004)
Supplement: Multimedia Appendix 1 [file formative_v5i8e14004_app1.docx]

1. **What did you think of the app overall?**
   1. What bits of it were useful if any?
   2. What bits were not useful?
   3. What did you enjoy?
   4. What did you dislike?
2. What was it like using the app?
   1. What did you think of our avatar?
3. **How often did you use the app**?
   1. Was there a **particular time** you used the app? E.g. morning before school
   2. Was there **a particular place** you used to use the app? E.g. at school
4. **How could you make it better**?
   1. **Would you add new features**?
   2. **Would you do away with any features**?
   3. If you had unlimited money, what would you change?
5. **What did you think of the games**?
   1. How do the games compare to other games you might play on your phone?
   2. What would you like to see more of?
   3. What would you like to see less of?
6. **What did you think of the other features in the app**?
   1. What did you think of the questionnaires?
   2. What did you think of the goals?
   3. What did you think of the journal?
7. **How would you describe the app’s ability to help you with stress**?
   1. How would you describe the app to a friend?
8. **What do you think about technology in general?**
9. **What do you think about videogames?**
